# A Qualitative Examination of the Role of Small, Rural Worksites in Obesity Prevention

**Published:** 2011-06-15

**Authors:** Cam Escoffery, Michelle C. Kegler, Iris Alcantara, Mark Wilson, Karen Glanz

**Affiliations:** Department of Behavioral Sciences and Health Education, Rollins School of Public Health, Emory University; Rollins School of Public Health, Emory University, Atlanta, Georgia; Rollins School of Public Health, Emory University, Atlanta, Georgia; Langdale Industries, Inc, Valdosta, Georgia; University of Pennsylvania, Philadelphia, Pennsylvania

## Abstract

**Introduction:**

The prevalence of overweight and obesity in the United States is highest in rural counties. We explored social support, policies, and programmatic resources that encourage more healthful diets and participation in physical activity among employees of small, rural worksites.

**Methods:**

We conducted in-depth interviews with 33 employed adults aged 50 years or older in rural Georgia about access to healthful foods and opportunities for physical activity at work; conversations about exercise, weight loss, and eating healthfully in general; and worksite nutrition and physical activity programs; and we asked for suggestions for making the worksite more healthful. The research team developed a codebook, and 2 coders coded each transcript. Data were analyzed and reports were generated for thematic analyses.

**Results:**

Participants from rural worksites, most with fewer than 50 employees, cited lack of vending machines and cafeterias, health promotion programs to address healthful eating and exercise, and facilities for physical activity as barriers to eating healthfully and engaging in physical activity at work. Many participants reported conversations with coworkers about how to eat more healthfully by making more nutritious choices or preparing food more healthfully. Participants also discussed the importance of engaging in physical activity on their own and gave suggestions on ways to incorporate exercise into their routines. Participants' access to healthful foods at work varied, but barriers such as being too busy, worksite location, and no worksite cafeteria were noted. Some workers reported engaging in physical activity at work, and others reported a heavy workload and lack of time as barriers.

**Conclusion:**

Building on the social environment and implementing policies for healthful eating and participation in physical activity may help address obesity prevention in rural workplaces.

## Introduction

The prevalence of overweight and obesity in the United States continues to be a public health concern. In 2008, 32% of men and 35% of women in the United States were overweight or obese (1). Overweight and obesity are associated with increased risk for diabetes, stroke, heart disease, some types of cancers, hypertension, high cholesterol, and arthritis (2). Obesity prevalence is lowest in urban counties throughout the United States and highest in rural counties, particularly in southern states (3).

A growing body of research suggests that body weight is determined both by behaviors (eg, eating less, being more active) and environments (ie, social and built) (4-6). Worksites are practical settings for health promotion, and worksite health promotion can improve employee productivity and health, including playing a role in modest weight loss (7-9). Although results are mixed, worksite intervention studies aimed to improve dietary and physical activity habits of employees are promising when combined with strategies that also target social and organizational support, environmental change, and policy change (10-13).

However, despite the promise of worksite environments in supporting employees' healthful eating and participation in physical activity, small or rural businesses typically have fewer health promotion programs than larger employers (8,14-16). A recent national survey of worksites indicated that worksites with more than 750 employees consistently offered more health promotion programs than did smaller worksites (17). Small worksites face barriers such as lack of financial resources and infrastructure to conduct health promotion programs (8,14). Because more than 50% of workers are employed by businesses that have fewer than 100 employees, learning more about small and rural worksites and their potential for providing health-promoting environments and programs is important (14,16,17).

The purpose of this qualitative study was to learn more about the available intervention strategies and policy and social supports of worksites in rural areas. We examined the social support, policy, and programmatic resources in rural worksites that may encourage more healthful diets and participation in physical activity among their employees.

## Methods

The Emory Prevention Research Center (EPRC), its Community Advisory Board (CAB) of representatives from multiple community sectors, and the Southwest Georgia Cancer Coalition partnered to conduct this research. The CAB assisted with decisions about study design, the data collection instrument, and interpreting results. A Southwest Georgia Cancer Coalition staff member supervised data collection and the local residents trained in interviewing techniques by Emory faculty and staff who conducted the interviews. The Emory University institutional review board approved the research protocol.

### Sample and procedures

The study was conducted during May through September 2005. Eligibility requirements were being African American or white, aged 50 years or older, currently living with at least 1 other person, and residing in the Georgia counties of Calhoun or Terrell for at least 10 years. Calhoun (population 6,094) and Terrell (population 10,657) counties are located in rural, southwest Georgia and are characterized by high rates of poverty and low educational attainment (18). We employed a snowball sampling approach to recruit 60 participants, divided evenly by sex and race. The CAB decided to focus on adults aged 50 or older because members thought that prevention of cancer and other chronic diseases would be more salient for adults in this age range.

Interviewers recruited participants through snowball sampling, starting with personal contacts, local businesses, and organizations. They were also recruited by going door-to-door in neighborhoods and at civic associations and by using advertisements in local papers. Interviewers screened potential participants in person to ascertain eligibility, inquiring about age, living with another person, and time lived in Calhoun or Terrell counties. Sixty participants were interviewed in their homes or public areas, and all provided written informed consent. All interviewers attended a 1.5-day training about the project and qualitative interviewing methods. They completed several pilot interviews and received feedback on their technique. The trained local residents conducted the interviews using a semistructured interview guide; they were race- and sex-matched to respondents.

### Instrument

The full interview guide explored how social and physical environments in the home, work, and church influence healthful eating, participation in physical activity, and tobacco use. Only themes regarding how the worksite affects healthful eating and participation in physical activity are reported here. Data on the role of the home and neighborhood environments on these behaviors have been published elsewhere (19).

Questions about worksite environments that promoted healthful eating and weight loss asked about the availability of a cafeteria and vending machines, discussions with coworkers about eating healthfully and losing weight, and availability of worksite programs to help people eat healthfully or lose weight (Appendix). Questions about physical activity environments and opportunities asked about participating in physical activity at work, conversations with coworkers about participating in physical activity, and the availability of worksite programs or facilities for participating in physical activity. We also asked participants to offer suggestions on how to improve their worksites. The interviews typically took 60 minutes to complete. Interviewers gave participants a $20 gift card from Walmart as compensation for their time.

### Data analysis

The interviews were transcribed verbatim. We excluded 2 interviews in the final analysis because of tape-recording problems. The research team developed a codebook to cover major themes for each topic covered in the interview discussions. Two coders then coded each transcript independently and resolved discrepancies through consensus. QSR-N6 software (QSR International, Cambridge, Massachusetts) was used for data storage, retrieval, and analysis. The coders generated N6 reports with all comments associated with particular codes. Content analysis was performed to identify the range of responses and major themes (20). To identify patterns and themes by sex and race, matrices were constructed (21,22). For example, staff generated a report to retrieve all text associated with availability of healthful food choices at work by sex and race. One researcher identified themes and a second confirmed the themes (eg, vending or cafeteria options). The researchers defined strength of a theme by number of responses: 5 or fewer responses being weak, 6 to 14 responses being moderate, 15 responses or more being strong.

## Results

Of the 58 respondents, 33 (57%) reported being currently employed outside of the home. Of these 33, most were white men and had a mean age of 59 years (Table 1). Most worksites employed 50 or fewer people; 16 participants reported working with 4 or fewer people. Many participants worked in small retail stores and offices (eg, art shop, florist, bookkeeping), and others worked at factories, in construction or education, or were self-employed (eg, landscaper, computer repairman).

### Healthful eating at work

**Opportunities for healthful eating at work**

**Cafeterias and vending machines.** Two strong themes that emerged were the lack of cafeterias and vending machines in these rural worksites. Approximately half of respondents reported that cafeterias were not present at their worksites. Those participants whose worksites had cafeterias described prepared meals, meats and fish, sandwiches, side items, and desserts as being generally available:

When we had the meal, I had the vegetables and all, but then they quit cooking anything but the chicken and chicken tenders and so I wound up eating chicken tenders. I would make me a chicken tender sandwich, and that was fried and it was not good for me. (white female participant)

A little more than half of respondents reported that vending machines were not present at their worksites. Of participants whose worksites had vending machines, only 2 stated that they purchased food daily from a vending machine. Vending machines typically offered food such as snacks (eg, candy, chips), beverages, and occasionally salads and sandwiches:

They have these vending machines and you know they have sandwiches, and sometimes they put salads in there. . . . No fruits, they used to put apples in them but none of them do now. . . . Well, I buy something [like a] sandwich made with brown bread [or] a salad, you know, a green salad. (African American female participant)

**Food from home.** About half of the respondents reported seldom or never bringing food with them from home to eat at work. The most common explanation for this was that participants usually went home to eat lunch (moderate theme). Other reasons mentioned by a few participants were lack of time in the morning, the convenience of eating out or eating at the worksite cafeteria, or only eating once per day:

Of course, I could bring stuff from home, but we have to be at work at 7:45 am and so to get up and fix food to bring, you know, it's a trade-off between a little more sleep or fixing food. (white female participant)

We eat at the China Berry café most every day. It's right next door, so . . . you know it's convenient and it's easy to go down there and eat. (white male participant)

A few respondents mentioned reasons for bringing food to work, which included not being able to leave work during the day to get food, the convenience of bringing food or snacks with them to work, and concern for the nutritional quality of the foods they eat:

It usually has to do with tight schedules. Like, when we're shipping something and I just can't leave because I don't know when the truck's going to pull up. (white male participant)

A couple of respondents mentioned that refrigerators or microwaves were available for them to store and prepare food at their worksite:

We do have a refrigerator that soft drinks and water are kept in, but other than that. . . . We do have a microwave where people bring in [food to reheat]. (white male participant)

**Eating out on a work day.** Approximately half of all respondents reported never or rarely going out to eat during the work day. The rest reported typically going out to eat 1 or 2 times per week, and 2 respondents said that they eat out every day. Respondents were divided on whether the meals eaten out were healthful:

I want to say pretty health[ful]. Now they . . . usually have 3 vegetables and 1 meat. . . . We have salads with tomatoes and then the vegetables, and then they also have bread, which is usually cornbread or something like cornbread. (white male participant)

They . . . cook soul food, butter beans, and chicken. . . . Don't say nothing about their broccoli casserole [laughing]. It's bad, and they cook breakfast. . . . Sometimes we buy breakfast. (African American female participant)

**Conversations with coworkers about healthful eating and weight loss**

Two strong themes that surfaced were conversations about eating healthfully and losing weight with colleagues at work ( Table 2). Most respondents (n = 17) reported having conversations with coworkers about eating healthfully, with the exception of African American male participants. Moderate themes from the conversations centered on the types of food participants eat (healthful or unhealthful) and methods to eat healthfully. Weaker themes were the methods participants use to prepare foods and discouraging coworkers from eating unhealthful foods.

Many conversations about losing weight at the worksite involved 1 coworker offering advice to other coworkers on how to lose weight, with exercise mentioned most often as a moderate theme. Participants also discussed conversations about eating healthfully to lose weight. Strategies mentioned were using weight-loss programs (eg, Weight Watchers), eating prepackaged meals (eg, Lean Cuisine, Healthy Choice) and eating home-cooked meals for lunch.

**Barriers to healthful eating at work**

More than half of respondents identified barriers to healthful eating at their worksite. One moderate theme that emerged was the lack of time. Other barriers that emerged as weak themes were the presence of "tempting" foods, limited selection of healthful food options at worksites, job stress leading to eating unhealthful foods, and location of worksites, which could limit access to healthful foods.

Participants who reported no barriers to eating healthfully at work had flexible schedules, which allowed them more choices to eat healthful lunches, and others felt it was easy to bring in healthful lunches or snacks to eat throughout the day:

It's not anything wrong about the health [of] the place because you know you bring what you're going to bring. You know it's good for you to eat. You can bring it with you. (African American female participant)

Another strong theme was the lack of health programs. None of the respondents stated that there were currently programs at their worksites to facilitate healthful eating or weight loss. A few participants described programs that had been implemented in their worksites in previous years and commented that these programs had been unsuccessful.

**Suggestions for how worksites could encourage healthful eating**

Most respondents had suggestions for what worksites could do to encourage more healthful eating among employees. Suggestions that emerged as weak themes included providing more healthful food options, offering fresh fruits and vegetables, using insurance premiums as incentives, providing educational materials, and having a designated lunch break for all employees. A few participants reported that worksites could not do much to encourage healthful eating because of the small size of the work facility; the varying location of the worksite, which may be miles away from restaurants or stores; and the belief that eating healthfully is a personal responsibility and not something that should be determined or influenced by the worksite.

### Physical activity at work

**Opportunities for participating in physical activity at work**

Most respondents (n = 30) stated they are physically active at work. Strong themes included walking at work and performing activity required by the job. Approximately half of the respondents reported walking at work, and approximately half reported engaging in other types of physical labor, including lifting boxes or books, climbing ladders or stairs, loading trucks, hauling water, painting, sweeping, shoveling, landscaping, working on a farm, doing laundry, squatting, and operating power tools:

Walking from place to place and spot to spot and pressing them clothes . . . I get plenty of exercise on my job. (African American female participant)

Well, I think we're physically active on the job, and all my employees are physically active because not many . . . sits down to work. Everybody stands up or [is] just moving around, you know . . . so there's a pretty good bit of physical activity inside of the grocery store even though it's not planned that way. (white male participant)

The few respondents who said they were not physically active at work explained that their job was sedentary in nature (ie, involved sitting at a desk for most of the day). A few respondents noted that physical activity should be completed during personal time outside of the workplace.

**Conversations with coworkers about physical activity**

Approximately half of the respondents described having conversations with coworkers about physical activity. Common topics included sharing their own exercise practices (moderate theme) and encouraging one another to exercise (weak theme). A few respondents talked about how age had affected their physical activity level (Table 3).

**Facilities and programs to encourage participation in physical activity at work**

Almost all of the respondents reported no exercise facilities were provided at work, a strong theme. Two of the 3 exceptions were teachers who mentioned having access to a gym and a track. None of the respondents reported having exercise programs at their worksites, though a few informal activities existed. Two respondents reported that coworkers organized walking groups with other coworkers at the worksite. A few respondents reported that employees take breaks during the work day to walk outside.

**Barriers to physical activity at the worksite**

Slightly more than half of the respondents felt there were barriers to being physically active at work. Moderate themes were the sedentary nature of the job or a schedule that does not permit exercise during the work day, because of heavy workload or irregular hours. Limited space for exercise was also mentioned as a barrier.

**Suggestions for how worksites could encourage participation in physical activity**

When asked, almost half of the respondents had no suggestions for what their worksite could do to help employees be more physically active. Of those who had suggestions, a few said that allotting time to employees to exercise during the workday would be encouraging, and others suggested that employers provide exercise equipment or designate an area for employee exercise. A few others said that it was not necessary because their jobs were already physically demanding.

## Discussion

This study was a qualitative examination of programs and social support for physical activity and healthy eating in rural, and primarily small, worksites. Other studies of worksite health promotion have been quantitative and have focused on large businesses (23,24). Findings from this study were unique. Specifically, our results indicate that small, rural worksites tend not to have vending machines and cafeterias, have more collegial support for healthful behaviors, and have almost no established health promotion programs.

Strong themes that emerged were respondents having conversations with coworkers about eating healthfully, losing weight, and engaging in physical activity. Small worksites may provide a greater sense of community and may offer a supportive social environment for behavior change through colleagues or groups (14,16). Similarly, Tessaro and colleagues found that women in rural worksites believed that social support in the workplace could facilitate behavior change for healthful living (25). We also found that, as previously reported, some employees in these settings are already physically active on the job; therefore, health promotion in these types of worksites could focus on leisure-time physical activity or other health issues (26). Social support may be important for promoting health behaviors in smaller worksites by changing social norms and company culture for healthful lifestyles and by offering models and supports for behavior change (8,27).

The strong theme of limited or no cafeteria options or vending machines in rural worksites is in contrast to larger worksites. A major barrier to healthful eating was limited selection of healthful options and greater presence of unhealthful foods. Some comments focused on the unhealthfulness of Southern-prepared meals such as fried foods and sugary desserts. Expanding healthful food options or increasing their visibility and offering healthful options in vending machines can influence healthful eating in larger worksites (11). However, other methods such as offering an opportunity to buy locally grown produce at work (28) or educating employees about healthful eating at home, since many participants in our study reported going home for lunch, may work better for rural businesses. Furthermore, respondents were interested in education, reminders, and incentives to encourage employees to eat more healthfully. These educational and policy actions taken by employers can promote a more healthful worksite (14).

Lack of facilities for physical activity at these rural worksites was a strong theme, which is consistent with previous research (16,29). Other reported barriers to engaging in physical activity at work, which emerged as moderate themes, were limited space, schedules, and heavy workloads. Suggestions were made for provision of equipment or allotment of time for physical activity at the worksite. Worksites can provide opportunities for exercise through policies of flexible schedules and breaks, strategies that promote physical activity (12). Worksites can offer enhanced opportunities for physical activity by changing the local environment through creating walking routes, providing exercise equipment, or providing access to existing nearby facilities (30). Easier solutions for small worksites may include creating walking routes around facilities such as parking lots or arranging for services at nearby fitness facilities.

Similar to previous studies in small businesses (16,24), respondents reported that their businesses offered few to no health promotion programs for nutrition or physical activity. Several strategies to reduce this barrier are for businesses to pool costs or programs, refer employees to community sources for services, and provide information from government and professional groups, which are available for no or low cost (16). Additional resources for wellness programs in rural areas are community organizations, community centers, schools, or churches.

Our study has several limitations. The sample of respondents may not be representative of workers in other rural areas. We did not validate respondents' reports of the presence of worksite cafeterias, vending machines, and program offerings by conducting on-site visits. Respondents who reported being active at work may not meet the amount and type of activity recommended by national guidelines. Furthermore, we asked respondents to answer questions about their workplace offerings to glean insight into rural worksites; the reported availability of healthful foods, programs, and facilities varied on the basis of worksite setting.

Given the fact that more than 55% of Americans work for employers with fewer than 100 employees (15), access to worksite health promotion is critical. Future research could systematically assess the differences in policies that are health promoting at worksites of varied sizes and the effectiveness of social support programs, policies, and environmental changes on healthful eating and participation in physical activity at rural worksites.

## Figures and Tables

**Table 1:** Demographics of Rural Workers in Calhoun and Terrell Counties, Georgia, 2005

**Characteristic^a^**	No. of Men (n = 19)	No. of Women (n = 14)	Total No. (N = 33)
**Race**			
White	11	7	18
African American	8	7	15
**Age,^b^ y**			
50-59	11	8	19
≥60	8	6	14
**Education**			
High school graduate or less	6	8	14
Some college or college graduate	12	6	18
**Annual household income, $**			
<25,000	6	4	10
≥25,000	10	8	18
**Marital status**			
Married	15	10	25
Other^c^	3	4	7
**County of residence**			
Calhoun	3	4	7
Terrell	16	10	26
**Workplace size**			
≤4	9	7	16
5-49	8	5	13
50-199	2	2	4

a Numbers may not sum to totals for n because of missing data.

b Mean age for men, 59.1 years (standard deviation [SD] = 7.8 y); mean age for women, 60.1 years (SD = 6.5 y); and mean age for total sample, 59.5 years (SD = 7.2 y).

c Living with someone, divorced or separated, single, or widowed.

**Table 2:** Comments Related to Healthful Eating, by Topic and Theme, Among Employees of Small Worksites in Rural Georgia, 2005

**Theme/Topic**	Participant Comment
**Conversations About Healthful Eating at Work**	
Commonly discussed	. . . I think in any working environment where it's a small group, there's that interaction about food constantly. I mean that's a pretty good topic for people to talk about. (white male participant)
Types of food	Occasionally we talk [about] why we don't eat this particular thing because it has "x" number of calories or we don't need this . . . and we talk about eating fruit, because you need fruit [more] than that other stuff. (African American female participant)
Food preparation	Yes . . . [we] talk about how they're preparing food and what kind of foods they're preparing. . . . I'll bring a sample of something that we had for dinner back to work . . . then talk to them about how they're cooking their foods. They're pretty much meat and potatoes–oriented, you know. Try to expand that a little bit. (white male participant)
	Well, we'll just talk about the need to have less fat or how greasy the food is in the lunch room and how we wish they'd drain the stuff better, at least, that sort of thing . . . and the fact that they tend to empty the salt shaker into the food. (white female participant)
Programs for healthful eating at work	It has none. In the past . . . they paid some lip service to that. At one point, they got some kind of grant — they being the administration I guess — they got some type of grant, and they actually bought some exercise equipment, which was supposed to go in the teachers' [lounge]. I don't know when they thought they were going to use it . . . but that kind of petered out, and I don't even know what happened to the equipment. . . . We had a couple of speakers one time several years ago talking to the teachers about that sort of thing, lifestyle, healthy lifestyles. (white female participant)
**Conversations About Losing Weight at Work**	
Physical activity	I've hired a couple of new men, and both of them are slightly overweight and I've talked to them about . . . losing weight and walking to work. . . . [One employee] has started walking to work. He lives approximately a half mile from the station. (white male participant)
Eating choices	[A coworker] lost a lot of weight and then he got married, and now he's gaining a little weight . . . so he talks [about how] he'll eat his Healthy Choice at lunch sometimes, and I don't know what he eats when he goes home. But yeah, it's a big conversation down here about losing weight. (white female participant)
**Barriers to Healthful Eating at Work**	
Lack of time	You're on the go. You really don't have time, so you're going to grab something that's quick and easy, and it's never healthy. Like, you know, run through McDonald's on the way to taking a load of concrete out, you know, just to not be hungry. You can get in there and be out in a minute or 2. (white female participant)
Presence of unhealthful food	The presence of the food on Friday makes it hard because it's tempting . . . cinnamon buns and so forth. That's a real treat. (white male participant)
Lack of access to healthful food	Well, first of all, they don't have no healthy food on the job. . . . No, they don't have anything but them vending machines, you know, and there isn't anything in them but snacks. (African American male participant)
	Like I said, where you work, the place you working at . . . [is] way out in the country somewhere or somewhere not close to a store or a restaurant, so you have to say, "Go with what you got." Or . . . somebody might go and get a lunch for everybody, but you still have to buy that. (African American male participant)

**Table 3:** Comments Related to Physical Activity, by Topic and Theme, Among Employees of Small Worksites in Rural Georgia, 2005

**Theme/Topic**	Participant Comment
**Conversations About Physical Activity at Work**	
Talking about own exercise efforts	The lady I work with tries to walk, but it's very difficult for her in the heat . . . so that knocks out summer, and then when it gets too cold, so she really doesn't have much time, does she? [The conversations] are usually about her walking and just what she's doing, not what I do, she knows what I do. (white female participant)
Encouraging each other to be physically active	Oh, we talk about it all the time, and we try to encourage each other, "I'm going to go home and I'm going to walk 2 miles," or whatever. Or, I should, yeah, [or we should] because she's very health conscious, too. (white female participant)
Age affecting physical activity	We talk [about] that we cannot do what we used to could do (laughing). (white female participant)
	Oh, we talk a lot of times about being physically active, what we're going to do today, what we're going to go down there to the Sandtrap at night or House of Jazz and stuff. I be just bullshitting . . . they're not going to go down there. Man, I can't, I used to dance pretty good. I can still step before 2 men now. . . . If I do get out there I'm huffing and puffing, I don't want them to know nothing about it. (African American male participant)
Programs for physical activity for work	Exercise like I'm going to tell you right now, we'll exercise sometime about twice a week, and we'll walk 20 minutes on the inside, around and around there, the whole group. (African American male participant)
	. . . and sometime I will take a long walk around the plant, you know just to be going around the plant. I got that idea from an employee. (African American female participant)
**Barriers to Physical Activity at Work**	
Sedentary nature of the job	Well, a lot of it's involved in standing behind the counter checking out books, or sitting at a desk cataloging books, and the only physical part is the shelving. (white female participant)
Workload	At lunchtime or after the children had left from school but usually I had things I needed to do to prepare for the next day, so while I was at school I tried to do what I needed to do. (white female participant)
Irregular schedule	The long hours as it pertains to me, being a business owner . . . I don't come at 8:00 am and leave at 5:00 pm. I might come in at 7:00 am one morning and not leave till after midnight the same day. In fact one day this past Wednesday I was at work about 8:00 am, 8:15 am, on Tuesday morning and I didn't get home. . . . I stayed up all night Tuesday night and didn't get home the following night, Wednesday night, till 9:30 pm. (white male participant)
Limited space	Because you're confined to your one little office to do your job that you're sitting down at. (white female participant)
**Suggestions About Increasing Physical Activity for Worksites**	
Allot time for exercise	Probably just setting aside the time for it, you know, allowing a time within the work day to take a break and do that. (white male participant)
	Give you time and space, give you time to do things like that. (African American female participant)
Provide space for exercise	Well, if we had the space, maybe a place to maybe do yoga and that sort of thing at lunchtime, my daughter does that in her workplace . . . they enjoy it. (white female participant)

## Appendix. Interview Questions and Probes Related to Rural Worksite Health Promotion, by Topic, Georgia, 2005

**Food Availability at Worksite**

Is there a cafeteria or food service where you work?
How often do you buy food there?
*What do you usually buy?*
*Do they sell healthful foods?*
*What kinds of healthful foods?*
*Do they sell fruits and vegetables?*
Are there vending machines where you work?
How often do you buy foods from the vending machines?
*What do you usually buy?*
*Do they sell healthful foods?*
*What kinds of healthful foods?*
*Do they sell fresh fruits?*

**Food Brought From Home/Eating Out on a Work Day**

How often do you bring food from home to eat at work?
What kinds of foods do you typically bring?
How often?

**Conversations With Coworkers About Healthful Eating, Weight Loss, and Physical Activity**

Do you and your coworkers ever talk about eating healthfully?
Can you tell me about one of those conversations?
Do you and your coworkers talk about losing weight?
Can you tell me about one of those conversations?
Do you and your coworkers ever talk about being physically active?

**Opportunities for Physical Activity at Work**

What types of physical activity, if any, do you do on the job?
What exercise or recreation facilities, such as gymnasiums or outdoor fields, does your worksite have, if any?
Tell me about any programs your worksite might have to encourage people to be physically active.

**Suggestions for How Worksites Could Encourage Healthful Eating and Participation in Physical Activity**

What about your job or your workplace makes it hard to eat healthfully?
Given that, what are the top couple of things your workplace could do to encourage you to eat healthfully at work?
What about your workplace makes it hard for you to be physically active?
What are the top couple of things your workplace could do to encourage you to be physically active?
